# Effectiveness of GP intervention on risk drinking clients: a re-analysis of data of the Florence early identification and brief intervention project

**DOI:** 10.1186/1940-0640-8-S1-A22

**Published:** 2013-09-04

**Authors:** Manuele Falcone, Pasquale Pepe, Fabio Voller, Allaman Allamani

**Affiliations:** 1 Italian Health Agency, Tuscany Region, Florence, Italy

## 

Although implementing brief intervention among risky clients by general practitioners (GPs) is part of public health efforts, a greater challenge is to evaluate the impact of such intervention. The authors re-examined the data elicited by a GP brief intervention project, which involved 308 risky drinkers among 2869 clients enrolled between 2005 and 2007 in two areas in and around Florence. Data presented in previous INEBRIA meetings showed that baseline risky drinkers had a 55.30 g/day average intake (60.15 g for males, 41.63 g for females), which decreased to 37.61 g/day at the first follow-up (41.71 g for males, 26.06 g for females). Regression to the mean methods (both uni- and multi-variate) were used to overcome possible statistical flaws. Applying the regression coefficient of the two series of measurements, the extent of regression to the mean was 6.77 g and 6.78 g for males and females. Therefore, the reduction of the consumption in grams between enrolment and the first follow-up has been transformed respectively into 11.67 and 8.80 g/day. This change does not appreciably alter the previous results. The GPs who identify between 8% and 20% of their patients as at-risk drinkers (which is the rate considered as more approximate to reality) had a statistically significantly higher number of patients showing up at follow-up (68.69%) compared to those who identify a lower (<8%) or higher rate of clients (> 20 %) (p <0.001). Women compared to men, and people aged 40-49 compared to the other age classes, appear to be most amenable to change. Identification of risky drinking by GPs is a good predictor of decreased alcohol consumption among clients. Being a woman and being middle-aged are good predictors of improved alcohol drinking patterns among clients with at-risk drinking.

